# Randomised Trial of Planned Caesarean Section Prior to Versus after 39 Weeks: Unscheduled Deliveries and Facility Logistics - A Secondary Analysis

**DOI:** 10.1371/journal.pone.0084744

**Published:** 2013-12-20

**Authors:** Julie Glavind, Tine Brink Henriksen, Sara Fevre Kindberg, Niels Uldbjerg

**Affiliations:** 1 Department of Obstetrics and Gynecology, Institute for Clinical Medicine, Aarhus University Hospital, Aarhus, Denmark; 2 Perinatal Epidemiology Research Unit, Institute for Clinical Medicine, Aarhus University Hospital, Aarhus, Denmark; 3 Department of Pediatrics, Institute for Clinical Medicine, Aarhus University Hospital, Aarhus, Denmark; Cardiff University, United Kingdom

## Abstract

**Objectives:**

To compare the impact of scheduling caesarean section prior to versus after 39 completed weeks of gestation on the occurrence of unscheduled caesarean section and rescheduling of the procedure.

**Methods:**

Secondary analysis from a multicentre randomised open-label trial including singleton pregnant women with a healthy foetus and a reliable due date. Women were allocated by computerized telephone randomisation to planned caesarean section at 38 weeks and three days or 39 weeks and three days. The outcomes were unscheduled deliveries with provided reasons, such as spontaneous labour onset or supervening complications, and any changes in the scheduled delivery date. Statistical analyses were according to intention-to-treat using Fisher’s exact test.

**Results:**

From March 2009 to June 2011 1,274 women were included. Median difference in gestational age at delivery was six days. Compared to the 38 weeks group, the women in the 39 weeks group were more likely to have an unscheduled caesarean section (15.2% vs. 9.3%; RR 1.64, 95% CI 1.21; 2.22), to deliver between 6 pm and 8 am (10 % vs. 6%; RR 1.68, 95% CI 1.14; 2.47), or to have the procedure rescheduled (36.7% vs. 23%; RR 1.6, 95% CI 1.34;1.90).

**Conclusions:**

Scheduling caesarean section after 39 weeks leads to a 60% increase in unscheduled caesarean sections and a 70% increase in delivery outside regular work hours as compared to scheduling of the procedure prior to 39 weeks.

**Trial Registration:**

**www.clinicaltrials.gov** NCT00835003 http://www.clinicaltrials.gov/ct2/show/NCT00835003?term=NCT00835003&rank=1

## Introduction

The number of studies recommending that elective deliveries should be performed after 39 gestational weeks has increased [1-7]. Nevertheless, elective deliveries prior to 39 weeks seem to continuously account for a large proportion of term caesarean sections, with suggested ranges from 29% to 57% [4,8-10]. One explanation for this circumstance may be uncertainty about the impact of late scheduling on the risk of having a non-elective procedure, i.e. due to labour onset, which may be of personal inconvenience or concern to the woman but also a challenge to the logistics and planning at the delivery facility. To avoid increasing the risk of an unscheduled caesarean section, it may in specific cases be considered to book the caesarean section prior to 39 gestational weeks. This could for instance be due to a long travel distance to the hospital, especially with fast progression of labour in a previous delivery, or with an expected difficult surgical procedure due to adherences. 

There is a lack of prospective, valid estimates of the association between scheduled gestational age and the risk of unscheduled procedures [11-12]. Physicians and health care providers responsible for delivery ward management need this information to provide proper planning and information to the women about to be scheduled for a planned caesarean section. 

In this paper we report the incidence of unscheduled and night-time procedures among women randomised to caesarean section prior to versus after 39 completed weeks (38 weeks and three days vs. 39 weeks and three days). We also report the incidence of caesarean sections that end up being rescheduled after booking of the procedure and evaluate the provided reasons for this.

## Materials and Methods

The protocol for this trial and supporting CONSORT checklist are available as supporting information; see Checklist S1 and Protocol S1. 

### Ethics Statement

Ethics approval was obtained from The Central Denmark Region Committees on Biomedical Research (ID M-20080142), and this approval was valid for all the participating hospitals. Verbal and written consent was obtained from all participants. The Danish Data Collection Agency (ID 2008-41-2522) approved collecting and handling of the data. 

The results presented in this paper were secondary outcomes from a randomised controlled open-label multicentre trial conducted in seven Danish tertiary hospitals from March 2009 to June 2011 [13]. Participants were enrolled when a caesarean section was planned in the prenatal clinic. The women were randomised to scheduling of the procedure eleven days before due date (38 weeks and three days ± two days, referred to as 38 weeks group) or four days before due date (39 weeks and three days ± two days, referred to as 39 weeks group). The study eligibility criteria have previously been described in detail [13]; participants were singleton pregnant women with a gestational age set by ultrasound prior to fifteen gestational weeks. Each participating hospital had a neonatal intensive care unit (NICU) and in-house obstetrician, paediatricians and anaesthesiologists available 24 hours a day, with physicians typically working in 8, 16 or 24 hours shifts. 

The randomisation procedure was performed using a computer generated voice response telephone randomisation with random block sizes of 2, 4, and 6. Furthermore, we stratified the women by centre and previous caesarean section. 

All study outcomes were obtained from individual patient records thirty days postpartum. Mode of delivery, date and time of delivery, and if delivery was performed at the scheduled date were registered. If an unscheduled caesarean section was carried out less than eight hours after the decision to deliver, the procedure was classified as unscheduled. Any rescheduling of the delivery date was registered within the following categories: Logistics (rescheduling due to emergency operations of other patients or insufficient staffing), contractions or rupture of membranes, woman´s request (rescheduled due to the woman´s wish without any medical or obstetric indication), supervening complications in mother or foetus (preeclampsia, vaginal bleeding, elevated liver enzymes, obstetric emergencies such as placental abruption, suspected uterine rupture, or any foetal condition warranting delivery), vaginal delivery (regardless of the reason why), or other reasons (miscalculation of the delivery date, information unavailable). 

The indications for caesarean section were categorized according to the information written on the study entry form. Maternal request was registered as indication when written on the study form, but also used as indication for women with one prior caesarean section and with no other indications listed.

A sample size of 1270 participants was calculated from estimated proportions of the primary outcome (neonatal admission within 48 hours of birth) of 8 versus 14 percent [13]. Basic demographic data were presented with counts and percentages for categorical variables, with mean and standard deviation for continuous, normally distributed variables, and with median (interquartile range; iqr) for continuous, non-normally distributed variables. We analysed all outcomes according to the intention-to-treat principle using a Chi-squared test (Fisher´s exact test with small cell counts) calculating relative risk (RR) and risk difference (RD) with 95% confidence intervals (CI) for dichotomous outcome variables. The statistical analyses were performed using Stata Statistical Software: Release 11 (College Station, TX: StataCorp LP).

## Results

**Figure 1:**
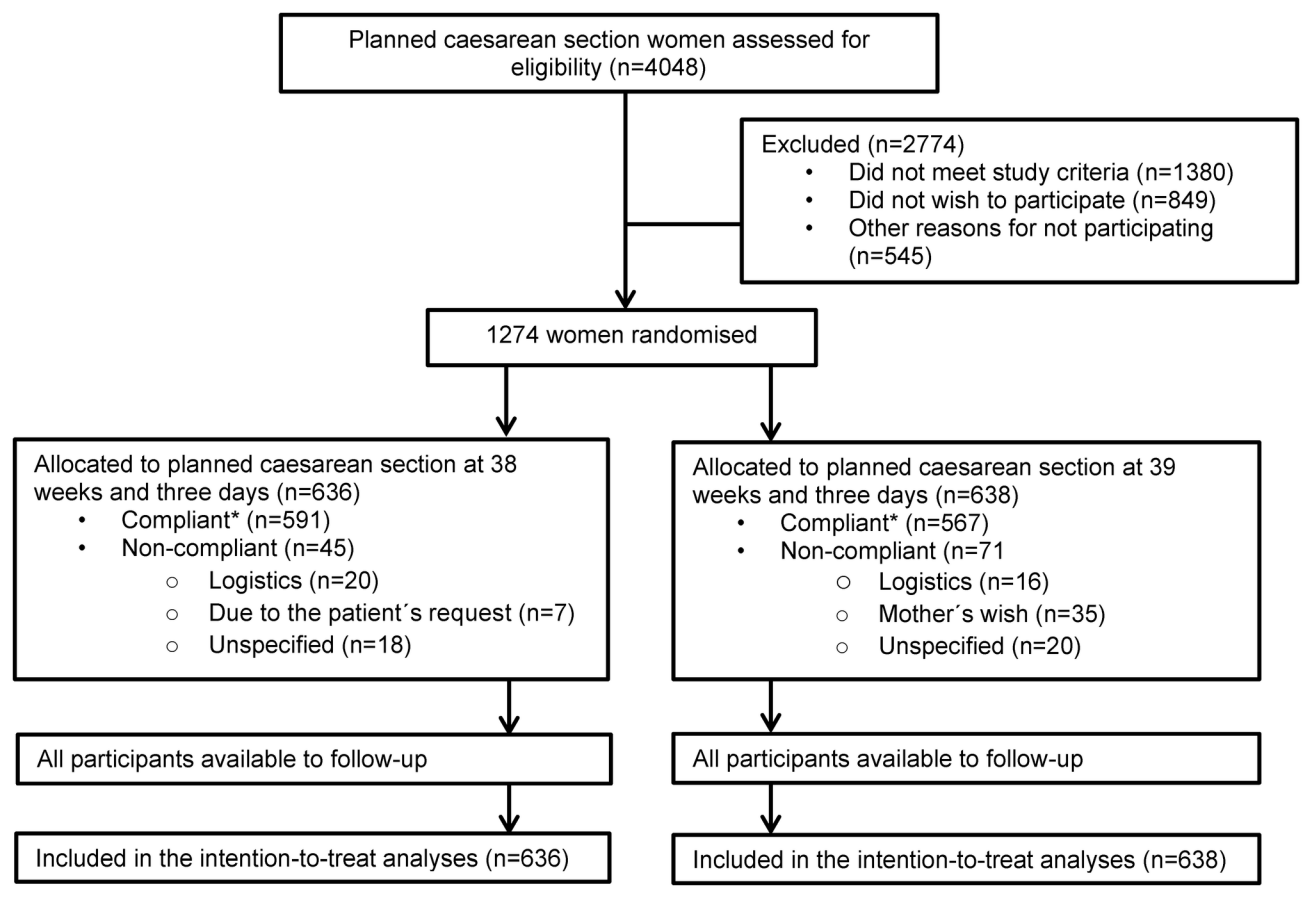
CONSORT flow chart. *Participants were defined as compliant if a caesarean section was performed within the randomisation group dates or at any other date due to labour or complications in pregnancy.

From March 2009 to June 2011 we enrolled a total of 1,274 women; 636 women in the 38 weeks group and 638 women in the 39 weeks group. All participants were available for follow-up and included in the intention-to-treat analysis (Figure 1). The women were included at a median gestational age of 33 weeks and four days (33 ^4/7^ weeks; range 12 ^3/7^ to 38 ^4/7^ weeks) in group 38 and 33 weeks and zero days (33 ^0/7^ weeks; range 11 ^4/7^ to 38 ^3/7^ weeks) in group 39, with 56.1% vs. 54.5% of the women in the two groups randomised after 32 completed weeks of gestation, respectively. Similar baseline characteristics were found in the two groups (Table 1).

**Table 1:** Baseline characteristics of trial participants with scheduling of caesarean section prior to versus after 39 weeks of gestation

	**Planned CS 39 weeks and three days** (N=638)	**Planned CS 38 weeks and three days** (N=636)
Maternal age in years, mean (SD)	31.6 (4.6)	32.1 (4.4)
Maternal height in cm, mean (SD)	167.6 (6.5)	167.7 (6.5)
Maternal weight in kg, median (iqr)	68 (49-87)	68 (50-86)
Body mass index ≥30 kg/m^2^	111 (17.4)	107 (16.8)
Maternal smoking	83 (13.0)	65 (10.2)
Gestational age validated by ultrasound before 15 weeks	636 (99.7)	636 (100)
Gestational diabetes at delivery	5 (0.8)	7 (1.1)
Nulliparous	117 (18.3)	126 (19.8)
Previous caesarean births		
0	255 (40.0)	253 (39.8)
≥1	383 (60.0)	383 (60.2)
Previous vaginal births		
0	421 (66.0)	426 (67.0)
≥1	217 (34.0)	210 (33.0)
Gestational age at randomisation		
<32 gestational weeks	290 (45.5)	279 (43.9)
≥32 gestational weeks	348 (54.5)	357 (56.1)

Data are presented as number (%) unless otherwise indicated. CS: Caesarean section, SD: Standard deviation, iqr: interquartile range

The participants were scheduled for caesarean section due to maternal request (41% vs. 48% in group 38 vs. 39, respectively), previous complicated delivery (12% vs. 13%), breech or transverse lie presentation of the foetus (18% vs. 14%), two or more prior caesarean sections (20% vs.16%), or planned caesarean section was advised by the physician or indicated due to maternal conditions (9% in both groups). 

Median gestational age at delivery was 38 weeks and three days in the 38 weeks group and 39 weeks and two days in the 39 weeks group. The proportion of unscheduled caesarean sections (Table 2) was significantly higher in women randomised to planned caesarean section at 39 weeks (15.2%) as compared to 38 weeks (9.3%) with a RR of 1.64 (95% CI 1.21;2.22, *p* = 0.001). More than 87% of unscheduled caesarean sections were performed due to spontaneous onset of labour. Planned caesarean section was less common in the 39 weeks group than in the 38 weeks group, RR 0.92 (95% CI 0.88;0.96, *p* < 0.001). More women in the 39 weeks group had a caesarean section between 6 pm and 8 am (RR 1.68, 95% CI 1.14; 2.47, *p* = 0.008).

**Table 2:** Urgency and time of day for caesarean sections with scheduling prior to versus after 39 weeks of gestation.

	**Planned CS 39 weeks and three days** (N=638)	**Planned CS 38 weeks and three days** (N=636)	**Relative Risk** (95% CI)	**Risk Difference** (95% CI)	***P***
Unscheduled CS	97 (15.2)	59 (9.3)	1.64 (1.21;2.22)	5.9% (2.3;9.5)	0.001
- labour onset	82 (12.8)	49 (7.7)	1.67 (1.19;2.34)	5.1% (1.8;8.5)	0.003
- maternal/foetal complications	15 (2.4)	10 (1.6)	1.50 (0.68;3.30)	0.8% (-0.7;2.3)	0.33
Planned CS	526 (82.4)	571 (89.8)	0.92 (0.88;0.96)	-7.3% (-11.1;-3.66)	<0.001
CS 6 pm - 8 am	64 (10)	38 (6)	1.68 (1.14;2.47)	4.1% (1.1;7.0)	0.008

Data are presented as number (%) of total number of women in the group. CS: Caesarean section.

With regard to the proportion of rescheduled deliveries (Table 3), 37% of women in the 39 weeks group vs. 23% in the 38 weeks group had their delivery date changed. In total, only 55/380 (15%) of the rescheduled delivery dates were moved towards the due date, and the majority of these were due to logistic challenges at the delivery ward (i.e. other emergency procedures). The main reason for rescheduling was spontaneous onset of labour (12.9% in the 39 weeks group vs. 7.7% in the 38 weeks group; RR 1.67, 95% CI 1.19; 2.34, *p* = 0.003). Rescheduling to an earlier delivery date due to supervening complications in mother or foetus was more common the 39 weeks group (RR 2.0, 95% CI 1.16; 3.42, *p* = 0.01). In order to explore the severity of these complications, we calculated the number of pregnancies in each group within the following entities: preeclampsia, hypertension, elevated liver enzymes, abruption of the placenta, foetal growth restriction, or foetal distress. Twenty-seven pregnancies or babies in the 39 weeks group compared to seven pregnancies or babies in the 38 weeks group suffered from these complications (*p* = 0.0007, Fisher´s exact test). 

**Table 3:** Rescheduling of caesarean section dates with provided reasons with scheduling of the procedure prior to versus after 39 weeks of gestation.

	**Planned CS 39 weeks and three days** (N=638)	**Planned CS 38 weeks and three days** (N=636)	**Relative Risk** (95% CI)	**Risk Difference** (95% CI)	***P***
Any rescheduling	234 (36.7)	146 (23.0)	1.60 (1.34;1.90)	13.7% (8.8;18.7)	<0.001
Rescheduling to a later date	16 (2.5)	40 (6.3)	0.40 (0.23; 0.70)	-3.8% (-6.0; -1.5)	0.0001
Rescheduling to an earlier date	218 (34.2)	106 (16.7)	2.05 (1.67; 2.52)	17.5% (12.8; 22.2)	<0.0001
Logistics	38 (6.0%)	44 (6.9%)	0.86 (0.57;1.31)	-1.0% (-3.7;1.7)	0.48
Mother´s request	36 (5.6%)	8 (1.3%)	4.49 (2.10;9.57)	4.4% (2.4;6.4)	<0.001
Labour onset	82 (12.9%)	49 (7.7%)	1.67 (1.19;2.34)	5.1% (1.8;8.5)	0.003
Mother or child at risk	38 (6.0%)	19 (3.0%)	2.00 (1.16;3.42)	3.0% (0.71;5.2)	0.01
Vaginal delivery	15 (2.4%)	6 (0.9%)	2.49 (0.97;6.38)	1.4% (0.01;2.8)	0.076
Miscalculations/ other	25 (3.9%)	20 (3.1%)	1.25 (0.70;2.22)	0.8% (-0.3;2.8)	0.45

Data are presented as number (%) of total number of women in the group. CS: Caesarean section.

Vaginal delivery occurred in 2.4% vs. 0.9% of the participants in group 39 vs. 38, respectively (RR 2.49; 95% CI 0.97; 6.38, *p* = 0.076), either due to imminent delivery (five women in group 39) or due to spontaneous version to cephalic presentation/ change of preferred mode of delivery to vaginal (ten women in group 39 and four women in group 38). The remaining two women (one in each group) had induced vaginal delivery due to stillbirth.

## Discussion

In a total of 1,274 women booked for planned caesarean section significantly more women had an unscheduled caesarean or were delivered by a caesarean section outside ordinary work hours when the procedure was scheduled after as compared to prior to 39 weeks. In addition, our data suggested that 27% of the women scheduled after as compared to 13% scheduled prior to 39 weeks were rescheduled due to unpreventable factors such as labour onset or complications in mother or foetus while awaiting the scheduled date. 

The major strengths of this study were the use of a randomised design and the novel logistic information and perspectives to the consequences of caesarean section timing. We also provided new information regarding the women´s own request for rescheduling of planned caesarean as well as the impact on the risk of rescheduling of the caesarean section towards an earlier gestational age due to supervening complications in mother or foetus. 

Shortcomings of the study may be that the outcomes reported in this paper were all secondary trial outcomes and that the statistical power for evaluating the reported outcomes was not estimated *a priori*. 

Since women in the 39 weeks group continued their pregnancy for another six days (close to term) it would be expected that significantly more women in this group presented with labour onset prior to their scheduled delivery date. However, only 12.9% of the women had spontaneous labour onset in the 39 weeks group, which was somewhat lower than suggested from previous studies [1,2,11,14,15]. Neonatal respiratory morbidity has been shown to decrease if spontaneous onset of labour occurs prior to a caesarean section [16]. However, labour-related risks such as uterine rupture, neonatal infection, or procedural difficulties due to adherences may overrule any possible neonatal advantage from a labour-preceded caesarean section. In terms of maternal outcomes after caesarean section, adverse events are likely to increase with the urgency of the procedure [17,18] and with progression of labour [17,19,20]. A limitation to most previous studies is the inability to separate women with labour onset prior to a planned caesarean from women with unscheduled caesarean deliveries intended as vaginal. 

In this study there was a 10% (39 weeks group) versus 6% (38 weeks group) risk of operative delivery outside ordinary work hours. Gould et al. found a higher neonatal mortality rate with early and late night births as compared to daytime births [21], while Peled and colleagues found a similar risk of neonatal morbidity in deliveries during day and night time working shift [22]. Errors and poor judgment has been associated with physician sleep deprivation, but nevertheless the surgical performance seems to be sustained during night-shifts [23]. 

With a two-fold increase in risk of rescheduling due to complications, our results suggested that continuing pregnancy for six more days this close to term had significant impact on the risk of developing complications warranting an earlier delivery. Noticeably, this study reports the reasons for rescheduling rather than the actual impact on postpartum morbidity. We found no significant difference in neonatal admission within two days between the two groups, and the incidence of other neonatal and maternal short-term clinical outcomes were almost similar in the two intervention groups [13]. This may indicate that the excess of unscheduled procedures, night-time deliveries, or labour onset in the 39 weeks group did not translate into an overall increased risk to the neonate or mother.

In terms of external validity, the study participation criteria were defined in order to include only low risk women, and therefore we had no reason to believe that the mother or foetus would be put at risk when caesarean section was scheduled after 39 gestational weeks. Thus, the results may apply to women with a non-medical reason for a planned caesarean section and to women with an indicated caesarean section where timing prior to 39 weeks and five days would not be indicated [24]. Since nearly 95% of the women in this trial were white Europeans, scheduling caesarean section after 39 weeks may carry a larger risk of having an unscheduled procedure in populations with different ethnicities [25]. 

Both pregnant women and health facility administrators would benefit from the knowledge obtained in this study. With scheduling after 39 weeks the women should be informed of some 13% risk of labour onset prior to the scheduled delivery date, an overall 15% risk of having an unscheduled procedure, and a 27% risk of having her delivery date rescheduled due to unpreventable causes. In a delivery unit with 5000 births and an annual 10% planned caesarean section rate an additional unscheduled caesarean section would be performed every eighth night if 38 weeks and three days is the preferred timing policy and every fifth night if 39 weeks and three days scheduling is the policy. However, the estimates are modified by gestational age at booking.

In conclusion, scheduling a planned caesarean section at 39 weeks and three days as compared to 38 weeks and three days leads to a 60% increase in unscheduled caesarean sections and a 70% increase in delivery outside regular work hours, but also to a large increase in procedure rescheduling. Neonatal and maternal clinical outcomes and not logistics at the delivery facility or physician convenience should be the prevailing concern in timing of the procedures. Therefore, the results reported in this paper should be considered as practical information of the logistic consequences of scheduling planned caesarean section after 39 weeks and not as a recommendation of early term scheduling. 

## Supporting Information

Checklist S1 **CONSORT checklist.**
(DOC)

Protocol S1 **Trial Protocol.**
(DOC)
