# Supplementary material for: Randomised Trial of Planned Caesarean Section Prior to Versus after 39 Weeks: Unscheduled Deliveries and Facility Logistics - A Secondary Analysis
Source: PLoS One. 2013 Dec 20;8(12):e84744. doi: 10.1371/journal.pone.0084744 (PMC3869904; doi:10.1371/journal.pone.0084744)
Supplement: Protocol S1 — Trial Protocol. (DOC) [file pone.0084744.s002.doc]

**Timing of elective caesarean section and neonatal morbidity - a randomised multicentre trial.**

1. **Working Title**

Comparison of neonatal morbidity after elective caesarean section at 38+3 vs. 39+3 weeks: a randomised controlled multicentre study.

1. **Background**

Elective (planned) caesarean section accounted for approx. 10% of all births in Denmark in 2007. The caesarean section rate continues to rise globally (Villar et al.), which in part can be attributed to a rise in the number of caesarean sections performed without medical indications, i.e. upon maternal request.

Elective caesarean section (ECS) is scheduled prior to or at 39^+0^ gestational weeks at several Danish hospitals (Sandbjerg guidelines, personal communications). This is probably because the number of unscheduled or acute caesarean sections increases with scheduling closer to the due date due to an increase in women with spontaneous onset of labour prior to the scheduled date. It has been suggested that 10-17% of pregnant women will go into labour before 39^+0^ weeks (NICE, Hansen, Morrison). Previous studies have found an increased risk of maternal perioperative and postoperative complications with acute caesarean sections, but whether an increased risk also exists among women delivered by an acute, originally planned caesarean section is uncertain (van Ham, Allen).

Several observational studies reported an increased risk of neonatal respiratory morbidity with earlier term gestational ages (Hansen, Morrison, Zanardo). The incidence of neonatal admission after ECS without a medical indication may be 13.1% (Yee) or approximately 14% at 38^+0^-38^+6^ weeks, decreasing to 8% at 39^+0^-39^+6^ weeks (Hansen). Hansen et al. found a decreasing risk of respiratory morbidity in infants born by ECS from 37 to 39 weeks. Compared with neonates of the same gestational age delivered vaginally, the risk of respiratory morbidity was no longer significantly increased in neonates delivered after 39 weeks. No randomised studies have yet evaluated respiratory morbidity or complications requiring hospitalization of the neonate following ECS in the immediate pre-term weeks.

Admission to a neonatal ward is an evident burden, both for the family and from a health economic perspective. So is any complication from caesarean section suffered by the mother. This testifies to the need for a randomised study of the timing of term ECS to ensure optimum timing of caesarean section with minimal risk of complications for mother and child. In addition, such study would shed light on the important organizational aspect of optimizing the gynaecological ward and the maternity ward routines.

**3. Aim of study** 
The aim of this study is to compare ECS scheduled at 38^+3^ weeks and ECS scheduled at 39^+3^ weeks with respect to neonatal and maternal adverse events, with respect to organisational aspects of initially planned, unscheduled caesarean sections, and with respect to maternal satisfaction with timing of the procedure.

**3.1 Null hypotheses**

1. That there is no difference in neonatal morbidity measured as the incidence of NICU admission, infection and respiratory morbidity.
2. That there is no difference in infant morbidity (health care utilization) within the first two months and the first two years of life.
3. That there is similar maternal perioperative and postoperative morbidity between the two groups.
4. That women's satisfaction with timing of the ECS do not differ between the two groups.
5. That there is no difference in the incidence of maternal depression between the two groups.

**4. Method and materials** 
Open-label randomised multicentre trial. 
The pregnant women are assigned to one of two treatments: 

1) ECS at 38^+3^ weeks (range 38^+1^ to 38^+5^) 
2) ECS at 39^+3^ weeks (range 39^+1^ to 39^+5^) 

The pregnant woman is included at any stage of her pregnancy when planning ECS in consultation with her obstetrician. The obstetrician assesses her eligibility based on the inclusion and exclusion criteria. At the preliminary examination in the pre-natal clinic, the obstetrician fills in a worksheet to obtain the following information: Name, social security number, indication for caesarean section, date of confinement, age > 18 years, due date according to the nuchal translucency scan, singleton pregnancy, diabetes, if pregnancy may be continued to week 39+5, use of interpreter to communicate in Danish, smoking status, height, weight, and parity. Information on both participants and non-participants are collected.

The obstetrician provides oral and written project information to the woman and obtains written consent. The latter task may be performed by a project staff nurse or midwife.
Between seven and eight weeks after birth, an e-mail with a link to a questionnaire is sent to the participant who will be asked for permission to contact her at a later time in case any new research emanates from the material.

The pregnant woman will be excluded if she submits a request to this effect, and any change in the date of planned caesarean will be at the discretion of the local administrator. However, exclusion of the woman’s data from the study does not take place at any time after consent has been obtained as data are analysed by the intention-to-treat principle.

**4.1 Outcomes**

Primary outcome:

- Neonatal morbidity causing admission to neonatal ward within the first 2 days of life

Secondary outcomes:

1. Admission to neonatal unit within 7 days after birth
2. Neonatal length of stay and ICD-10 diagnoses for hospitalized children
3. Neonatal treatment and duration of treatment: Mechanical ventilation, CPAP, oxygen supplementation, intravenous antibiotics
4. Outpatient treatment or control, or admission to a neonatal unit or paediatric ward within 2 months or 2 years after birth
   1. Department or physician, date of admission and discharge, ICD-10 codes, treatment, type of medical treatment.
5. Maternal morbidity within 30 days
   1. Perioperative complications: Uterine incision lateral tear, blood loss, laceration of bladder or intestine, other intraoperative complications
   2. Postoperative complications: Blood transfusion, need of re-operation, antibiotic treatment, and total hospital stay.
6. Logistics
   1. Acute caesarean section (8 hours from decision to execution)
   2. Reason for acute caesarean section: labour, PROM, foetal indication, pregnancy complications, maternal request
   3. Time of delivery
   4. Education & training of operating physicians
7. Child’s nutritional status after 2 months
8. Mother’s preference for time of caesarean at any subsequent pregnancy.

**4.2 Randomisation** 
The due date is based on biometrics determined by nuchal translucency scan (at 12 weeks) with duration of pregnancy of 280 days. 
Randomisation is performed with an automatic computerized telephone system (Voice response) that meets international requirements. The randomisation is stratified by centre and previous caesarean section. 
According to the randomisation protocol, the obstetrician schedules the date of surgery in collaboration with the staff booking the ECS. The procedure is booked according to a prioritized gestational age sequence:

| EARLY ECS (38+3)   1. 38+3 2. 38+2 3. 38+4 4. 38+5 5. 38+1 | LATE ECS (39+3)   1. 39+3 2. 39+4 3. 39+2 4. 39+1 5. 39+5 |
| --- | --- |

**4.3 Blinding** 
The trial cannot be performed as a blinded study. 
The staff is instructed not to express any personal preferences in their interaction with the pregnant women. Open data analyses are carried out.

**4.4 Data collection**
Data are retrieved from the individual patient records and are entered into a study data registration form. 
Data regarding the child's progress within 2 months and 2 years after birth are retrieved either from medical records on the data registration form or are extracted from the Danish National Board of Health's national patient Register using the relevant personal security numbers (CPR). 
Data from the registration form are entered into EpiData by a secretary at the Perinatal Research Unit. Data from the questionnaire are downloaded from the server (Ramboll, SurveyXact). 
Data are kept confidential and in accordance with Danish Law. Obstetricians in charge of the study at each facility and project staff are responsible for correct data handling and storage. The project has been approved by the Danish Data Protection Agency.

**5. Power calculation**
The sample size is calculated on the basis of the primary endpoint of neonatal ward admission within the two days after birth, using data from an existing birth cohort from the same population. Approx. 14% of those delivered by early caesarean section and 8% of those delivered by late caesarean section are hospitalized. With a significance level of 5% and a power of 0.80, the calculated sample size is 459 in each group. With non-compliance of around 9%, the total number of enrolled participants is calculated to approx. 1,270 women.

**6. Project location and participants** 
Participants are recruited from Aarhus University Hospital Skejby, Hospital of Aalborg, Regional Hospitals of Herning, Viborg, and Randers, Kolding Hospital, and Odense University Hospital. The inclusion is initiated March 2009. 
An obstetrician at each hospital unit serves as local project manager (at Skejby the principal investigator). Each trial unit is compensated in the amount of DKK 700 for each participating woman, which corresponds to the time spent on inclusion, data collection, etc. All involved staff groups receive instruction as to the project’s background and practical aspects. 
The group of physicians perform caesarean section according to the joint Danish O&G professional guidelines (DSOG). All women receive prophylactic antibiotics intraoperative as well as thrombosis treatment (if indicated) after birth. A paediatrician is not routinely present at the ECS.

Around 2,235 children are delivered by caesarean section annually at the seven participating hospitals (2007). It is considered feasible to include 600 pregnant women annually. The inclusion period runs from 1 March 2009 and until the sample size is reached. From January 2011, the study only includes women with a due date earlier than 30 August 2011. This is expected to yield a total of approx. 1,300 included women (due to the restrictions on the due date in 2011). 
The study as a whole may be discontinued if it fails to recruit enough participants. As data are analysed in an open fashion and there are no plans for any interim analysis, we will not obtain information about significant differences in morbidity between the two groups that could cause the study to be terminated before it stops.

**Inclusion criteria**
• Scheduled for ECS at a hospital involved in the project 
• Legally competent person 
• Pregnant with one child 
• Due date determined by ultrasound before 15 weeks of gestation
 **Exclusion criteria**• Multiple pregnancy 
• Needs an interpreter to communicate in Danish 
• Diabetes, both before and in pregnancy 
• Where there is a risk that delivery will take place prior to 39^+5^ weeks

**7. Data processing and analyses** 
Data collected from the two groups are used in a comparative analysis to identify any inter-group differences on the specified parameters. 
Statistical variables will be calculated according to the intention-to-treat principle and per-protocol using the analytical software program STATA. The primary endpoint will be analysed with a 5% significance level. The budget foresees the need for a statistician to assist with the data analyses.

**8. Ethics** 
The study was approved by the Scientific Ethics Committee of the Central Denmark Region. 
In addition, the study had been adapted to the trial guidelines for Good Clinical Practice. 
The project has been reported to clinicaltrials.gov (ClinicalTrials.gov Identifier: NCT00835003).

**8.1 Side effects, risks, complications or discomforts from participation**
As mentioned above, several centres perform ECS around 1 week before the date of expected confinement, but the gestational age may vary by several days, e.g. if the centre only perform caesarean sections on a particular day of the week. Therefore, the potentially assigned dates of delivery are in many cases the same as if the women were not included in the project. 
ECS at an early date may potentially cause more respiratory complications in the neonate, whereas ECS at a late date may slightly augment the risk of having an emergency caesarean section, which result in more complications in the mother. Rather few of the project procedures will be scheduled at the extreme ends of group dates. With regard to the woman, she will have an operation that was already decided for. All issues related to the surgery and post-operative treatment will remain unchanged. 
The results of the project are expected to outweigh any minimally increased risk of complications on the part of the individual project participant.

New knowledge on the health of pregnant women and neonates that may result from the project will benefit future women and neonates delivered by ECS.

**9. Funding** 
The project has received external funding from private and public funds. As of 1st Sept. 2010, the following grants had been received:
The Danish Research Council of Health and Illness 1,620,000 DKK (including overhead DKK 400,000), The Central Jutland Region’s Medical Research Foundation DKK 1,500,000 (including overhead 45,000 million), Aase and Ejnar Danielsen Foundation 200,000 (including overhead 6,000), Sophus Jacobsen and Wife Astrid Jacobsen's Foundation DKK 100,000 (including overhead 3,000) and Maria Dorthea and Holger From, Haderslev Foundation DKK 20,000 (including overhead 600).

**10 Research plan** 
1 June 2008 - 1 January 2009: Information meetings, official approvals obtained and application for funding, preparing materials and website.

1 March 2009 - 30 august 2011: Inclusion of pregnant women. Participation in postgraduate courses. Ongoing data entry. 
1 Sept. 2011 - 1 November 2011: Data collection completed, last data entered. 
1 Dec. 2011 - 31 Dec. 2012: Data analysis and compilation of articles and PhD thesis.

**11. Responsibilities and project management**
The project is initiated and drafted by its steering committee.

The daily responsibility for project management is entrusted to PhD student Julie Glavind. 
Main supervisor and obstetrical professor in charge, professor, MD, DMSc, Niels Uldbjerg. 
Specialty supervisor, director of Perinatal Research Unit, head of the Neonatal Department, Professor, PhD, MD, Tine Brink Henriksen. 
Specialty supervisor with responsibility for organizational management and randomised trial, PhD. Clinical Midwife, Director, Sara F. Kindberg. 
All are affiliated with Aarhus University Hospital, Skejby.

**12. Results and publications** 
The results are presented in articles submitted to peer-reviewed international journals. The results will eventually be presented in a PhD thesis. A popular scientific presentation of the results may be obtained from the project website or by contacting the project manager.

**13. Perspectives**The main perspective lies in a clarification of the optimal timing of ECS from the perspective of obstetrics and neonatology and in the implementation into daily practice of the results of the study of organizational and health-related issues that emerge when scheduled caesarean sections turns into a sub-acute procedure.

References

Villar, J., Carroli, G., Zavaleta, N., Donner, A., Wojdyla, D., Faundes, A., Velazco, A., Bataglia, V., Langer, A., Narvaez, A., Valladares, E., Shah, A., Campodonico, L., Romero, M., Reynoso, S., de Padua, K.S., Giordano, D., Kublickas, M., & Acosta, A. 2007. Maternal and neonatal individual risks and benefits associated with Caesarean delivery: multicentre prospective study. *BMJ*, 335, (7628) 1025 available from: PM:17977819

Hansen, A.K., Wisborg, K., Uldbjerg, N., & Henriksen, T.B. 2008. Risk of respiratory morbidity in term infants delivered by elective Caesarean section: cohort study. *BMJ*, 336, (7635) 85-87 available from: PM:18077440

Morrison, J.J., Rennie, J.M., & Milton, P.J. 1995. Neonatal respiratory morbidity and mode of delivery at term: influence of timing of elective Caesarean section. *Br.J.Obstet.Gynaecol.*, 102, (2) 101-106 available from: PM:7756199

van Ham, M.A., van Dongen, P.W., & Mulder, J. 1997. Maternal consequences of Caesarean section. A retrospective study of intra-operative and postoperative maternal complications of Caesarean section during a 10-year period. *Eur.J.Obstet.Gynecol.Reprod.Biol.*, 74, (1) 1-6 available from: PM:9243191

Allen, V.M., O'Connell, C.M., & Baskett, T.F. 2006. Maternal morbidity associated with cesarean delivery without labor compared with induction of labor at term. *Obstet.Gynecol.*, 108, (2) 286-294 available from: PM:16880297

Hillan, E.M. 1995. Postoperative morbidity following Caesarean delivery. *J.Adv.Nurs.*, 22, (6) 1035-1042 available from: PM:8675855

Yee, W., Amin, H., & Wood, S. 2008. Elective cesarean delivery, neonatal intensive care unit admission, and neonatal respiratory distress. *Obstet.Gynecol.*, 111, (4) 823-828 available from: PM:18378740

Zanardo, V., Simbi, A.K., Franzoi, M., Solda, G., Salvadori, A., & Trevisanuto, D. 2004. Neonatal respiratory morbidity risk and mode of delivery at term: influence of timing of elective Caesarean delivery. *Acta Paediatr.*, 93, (5) 643-647 available from: PM:15174788

Hansen, A.K., Wisborg, K., Uldbjerg, N., & Henriksen, T.B. 2007. Elective Caesarean section and respiratory morbidity in the term and near-term neonate. *Acta Obstet.Gynecol.Scand.*, 86, (4) 389-394 available from: PM:17486457

van den Berg, A., van Elburg, R.M., van Geijn, H.P., & Fetter, W.P. 2001. Neonatal respiratory morbidity following elective Caesarean section in term infants. A 5-year retrospective study and a review of the literature. *Eur.J.Obstet.Gynecol.Reprod.Biol.*, 98, (1) 9-13 available from: PM:11516792

NICE guidelines (http://www.nice.org.uk/Guidance/CG13)
